# British Medical Association Meeting

**Published:** 1906-10

**Authors:** Dunbar Roy


### CORRESPONDENCE.

### BRITISH MEDICAL ASSOCIATION MEETING.

It was my good fortune to be complimented with an invitation to read a paper before the Ophthalmological Section of the British Medical Association, which met in Toronto, Canada, on August 20th of this year. The invitation was accepted and I am thus permitted to enjoy a most delightful outing, and at the same time meet our English friends across the border who are most courteous and attentive in making our visit one which will long be remembered. Thinking that the readers of the JOURNAL-RECORD would be interested in knowing just how our British colleagues manage their association, and in what ways they differed from our own, American Medical Association, I have taken the liberty of writing down some observations as they impressed themselves upon me, and which may not be without interest to others.

This was the second time that the Association had ever met in Canada, the other being in Montreal about ten years ago. The Association could certainly not have met in a more beautiful or more modern city than Toronto, and if other cities have entertained its guests in a more thorough or delightful manner than was done in this the capital city of Ontario Province, then such is beyond the imagination of the writer.

Toronto is the largest city in the Ontario Province, having about two hundred and fifty thousand inhabitants, and next to Montreal is the largest city in Canada. It is about four hours' ride from Buffalo by boat across Lake Ontario, and from its close proximity to the American line, it presents many of the features of a modern American town. The hotels are modern, and one or two compare favorably with the best in many of our American cities. The city is beautifully laid out, and the streets run with such regularity and arrangement that the stranger soon learns to guide himself unaided to the various points of interest.

But my remarks must be confined to the medical meeting. The British Medical Association was in session four days, and the meetings were held in the various buildings which constitute the University of Toronto. These latter consist of massive buildings of stone and brick, and together with the parliament buildings, are situated in an immense park, known as "Queen's Park," perhaps one of the most beautiful of its kind in Canada. The British Medical Association is constituted very much like our own American Medical, in that it is divided into various sections, representing every department of medicine, surgery and pathology, and each section is presided over by a president and usually four vice-presidents and three honorary secretaries. The large central building at the University was practically headquarters, as here was the place for all registration and information concerning all the meetings, and also numerous rooms where some of the section meetings were held, and where were also the exhibits, which constitute such a prominent place in all large medical gatherings at the present day. Other sections were held in various medical buildings situated on the campus, and in those buildings which were as a rule used for the work represented by the section. For instance, the sections in anatomy, physiology and pathology were held in the respective anatomical, physiological and pathological buildings, as also the section on practice, and in this manner practical demonstration could be given and the work of these sections rendered much more effective. This struck the writer as being most admirable, since the physicians in attendance could then obtain so much valuable information from these practical demonstrations which could not have been done if the meetings had been held at any other place. All the section meetings were held in the mornings, while in the afternoons the annual address and general meetings were held in what was called "Convocation Hall." This latter was an immense circular auditorium fashioned after the ancient Pantheon, and when completed will be a building with imposing features.

The annual address in medicine was delivered in the large auditorium on the afternoon of the second day, by Sir James Barr, M.D., F. R. C. P., his subject being "The Circulation Viewed from the Periphery"; and at eight o'clock the same evening Sir Victor Horsley delivered the address in surgery on the subject, "The Technique of Operation on the Central Nervous System." This latter was a most masterful and learned address, accompanied by numerous plates and illustrations. Sir Victor Horsley is perhaps the greatest living pioneer worker in this field of surgery, and it was indeed a great treat to hear this most famous neurologist and surgeon. During the afternoon meeting of the first day, at the installation of new officers and the president's annual address, Sir Victor Horsley was elected permanent vice-president of the Association. Mr. Horsley was quite a surprise to me in his personal appearance, since I had always looked upon him as a man of advanced years, probably due to my knowledge of his contributions to the physiology of the nervous system for so many years past, while in fact he did not look to be more than forty-five, tall and erect. Being interested in special branches, I only attended the section of ophthalmology, and that of laryngology. In both of these sections the men from the States, as they were designated, were very numerous, and took a very active part in the discussions. In fact, one saw almost as many doctors from the States as he did from Canada and the other British provinces. A point especially striking to me was the fact that the presiding officers of the sections were elderly men chosen for their ripened experience and knowledge of the subjects of their section, so that when one topic has been finished and discussed the president always summed up his own views on the subject and gave a resume of those most universally accepted by the best men everywhere, so that one had a very much clearer idea to take away after the topic was completed. The arrangement and management of this meeting was admirable for strangers. For instance, large signs were placed at intervals throughout Queen's Park directing you to any section you wished to attend, so that one experienced no difficulty in going just to the part he desired. The registration was in the large central building, and this was the only place where I found that they lacked system. In registering at the meeting of the A. M. A., one always found some eight or ten clerks, so that this part is accomplished with comparative celerity, but in Toronto there were only two clerks, and in addition, each man had to register on a separate page which, with a long waiting line, made this procedure rather tedious.

After getting your card and badge of registration, you passed to the opposite part of the room, where you secured cards to all the various society functions, of which there were many—about thirteen. Here also it lacked system, for the clerks in attendance were so polite as to go over the whole list of entertainments with you, and aid in picking out those which you would likely desire to attend. Consequently those behind you was a rather large and impatient crowd. Your badge and membership card served as an entré for you to all the various meetings, besides also being the card of admission to all the social and athletic clubs.

A rather unique feature to me was the publication each day of the "Daily Journal of the British Medical Association," which was a complete guide to all the meetings each day, the order of program for each section, the names of all physicians who had registered, as well as their addresses. This book also contained the names of all the officers of the Association, together with the various committees and the composition. In this way one felt that he knew each morning just what was to take place during the day, the hour and also the place. This is exceedingly helpful to one who is a stranger, and at the same time desires not to miss anything which is liable to take place. The English physicians are, as a rule, good talkers, and always to the point. Their accent was, of course, rather attractive and it was indeed delightful to hear the sonorous expressions which emanated from any group of talking Britishers. The Canadians have not quite so much brogue as the English, and this is largely due to their constant intercourse with the people from the States.

The Toronto General Hospital is one of the largest and oldest hospitals in Canada, and occupying as it does, a whole square, it has plenty of room for obtaining fresh air and furnishing outdoor comforts to convalescing patients. A very large afternoon reception was given at this hospital, and everything presented an air of decided neatness. The active presence of a magnificent band of music added much to the occasion, and we could readily see that it was a holiday occasion for both nurses and internes.

The Lord Mayor and General Council gave an evening reception at the city hall, and when we remember that such buildings in Canada are usually constructed of stone and marble, we can well expect a similar air of eloquence on the interior. Two magnificent bands discoursed music during the evening, and the presentation to the Lord Mayor and his wife, had all the dignity of a presentation at the court of King Edward. The typical Englishmen from across the water, and there were representatives from England, Scotland, Ireland, Australia, India, South Africa, Bermuda and all the British possession, were rather surprised at the medical progress in America, and one could readily see that both Canadians and those from the States, took pleasure in showing them what was being done on North American soil.

DUNBAR ROY, M.D.

---

### NOTICE TO MEDICAL PROFESSION.

At a meeting of the American Surgical Trade Association, held in Philadelphia, June, 1906, it was resolved that after January 1, 1907, the trade adopt the French scale for all catheters, bougies and sounds.

A committee was appointed for the purpose of getting up a proper and accurate French scale card, and the same will be mailed to you.

Every physician will see the importance of this step, as you are all acquainted with the annoyance of having catheters, bougies and sounds, and other instruments, marked in American, English or French numbers.

You are requested from above date to use only the French scale in ordering such goods, and when no scale is specified orders will be filled by the French scale. Your Dealer.

